# Hinchey Stage IV Diverticulitis in a Kratom User

**DOI:** 10.7759/cureus.90224

**Published:** 2025-08-16

**Authors:** Mitchell Stout, Emily Gioe, Mark H Smith

**Affiliations:** 1 General Surgery, Edward Via College of Osteopathic Medicine, Monroe, USA; 2 Medical School, Edward Via College of Osteopathic Medicine, Monroe, USA; 3 General Surgery, Surgery Critical Care, Willis Knighton Medical Center, Shreveport, USA

**Keywords:** bowel perforation, diverticulitis, hinchey stage iv diverticulitis, kratom addiction, kratom toxicity

## Abstract

Kratom is a partial mu-opioid receptor agonist that has gained popularity as an alternative to opioids for self-treatment of pain, anxiety, and opioid withdrawal. Despite its perceived safety, kratom use is associated with numerous adverse effects, including potentially life-threatening complications. We present the case of a 69-year-old male patient with a history of kratom abuse who developed Hinchey stage IV diverticulitis. Kratom’s dose-dependent effects, including gastrointestinal disturbances such as constipation, likely contributed to our patient’s condition. Understanding what kratom is, including its uses and potential risks, is essential for physicians to provide informed guidance, recognize kratom-related complications, and ensure patient safety.

## Introduction

Diverticulosis is one of the most common incidental findings on routine colonoscopy, affecting an estimated 60% of individuals by the age of 80 [1]. While many patients remain asymptomatic, 10-25% will develop diverticulitis [2]. The clinical presentation of diverticulitis varies widely, ranging from mild, uncomplicated disease to severe, and potentially life-threatening [3]. Complicated diverticulitis can be staged via computed tomography imaging using the Hinchey classification system. Stage I consists of a pericolic abscess or phlegmon; stage II includes a pelvic, intraabdominal, or retroperitoneal abscess; stage III is characterized by generalized purulent peritonitis; and stage IV presents with generalized fecal peritonitis [4]. The Hinchey system serves as a valuable tool for guiding management of patients with complicated diverticulitis [5]. We report a case of Hinchey stage IV diverticulitis in a kratom user.

Kratom is derived from the leaves of the *Mitragyna speciosa* tree, which is native to Southeast Asia and has been used by locals for centuries. Traditionally, the leaves were chewed, smoked, or brewed into tea for their stimulant and analgesic properties [6]. In recent years, kratom has gained popularity in the United States, with an estimated 2 million adults using it to self-treat pain, opioid withdrawal symptoms, anxiety, and depression [7]. The major active ingredients in kratom are the alkaloids mitragynine and 7-hydroxymitragynine, which act as a partial agonist at mu-opioid receptors and a competitive antagonist at kappa- and delta-opioid receptors [8]. Kratom’s effects are dose-dependent, producing stimulant-like activity at low doses and analgesic effects at higher doses [9]. Unlike traditional opioids, kratom’s partial mu-opioid receptor agonist activity is selective for the G-protein-coupled receptor pathway rather than the beta-arrestin signaling pathway, which is associated with many opioid-related adverse effects, such as respiratory depression [10]. This selective G-protein-coupled receptor activity likely contributes to kratom’s improved safety profile compared to other mu-opioid receptor agonists.

Although kratom may be a safer alternative to opioids, it still carries several risks and safety concerns. Reported side effects include tachycardia, drowsiness, vomiting, seizures, hallucinations, respiratory depression, and even death [11]. Kratom has also been found to inhibit gastrointestinal transit at high doses, leading to constipation [12]. Another major concern is kratom’s potential for addiction. Chronic use can result in cravings, tolerance, and withdrawal symptoms, particularly with frequent use [13]. Additionally, kratom interacts with various drugs, as it has been shown to inhibit several cytochrome P450 isotopes, including CYP3A, which metabolizes antidepressants, benzodiazepines, steroids, and tacrolimus [14]. At higher doses, kratom can lead to toxicity affecting multiple organ systems, including the liver, kidneys, lungs, heart, and central nervous system [15]. A Centers for Disease Control and Prevention (CDC) report identified 91 deaths where kratom was determined to be the cause between July 2016 and December 2017 alone [16]. However, the true scope of kratom-related toxicity is likely underreported, as most drug screens do not typically test for kratom, leading to unrecognized cases [17].

Despite its potential for abuse and toxicity, kratom remains unregulated at the federal level. As of February 2024, 22 states have enacted regulations on kratom, with Alabama, Arkansas, Rhode Island, Indiana, and Wisconsin making the sale of kratom illegal, while many states impose no restrictions [18]. The lack of regulation and the variety in kratom preparations raise concerns about accidental overuse. The potency of different kratom products can vary significantly; for example, traditional kratom leaves contain up to 2% mitragynine, whereas some kratom extracts contain up to 40% [19].

## Case presentation

**Figure 1:**
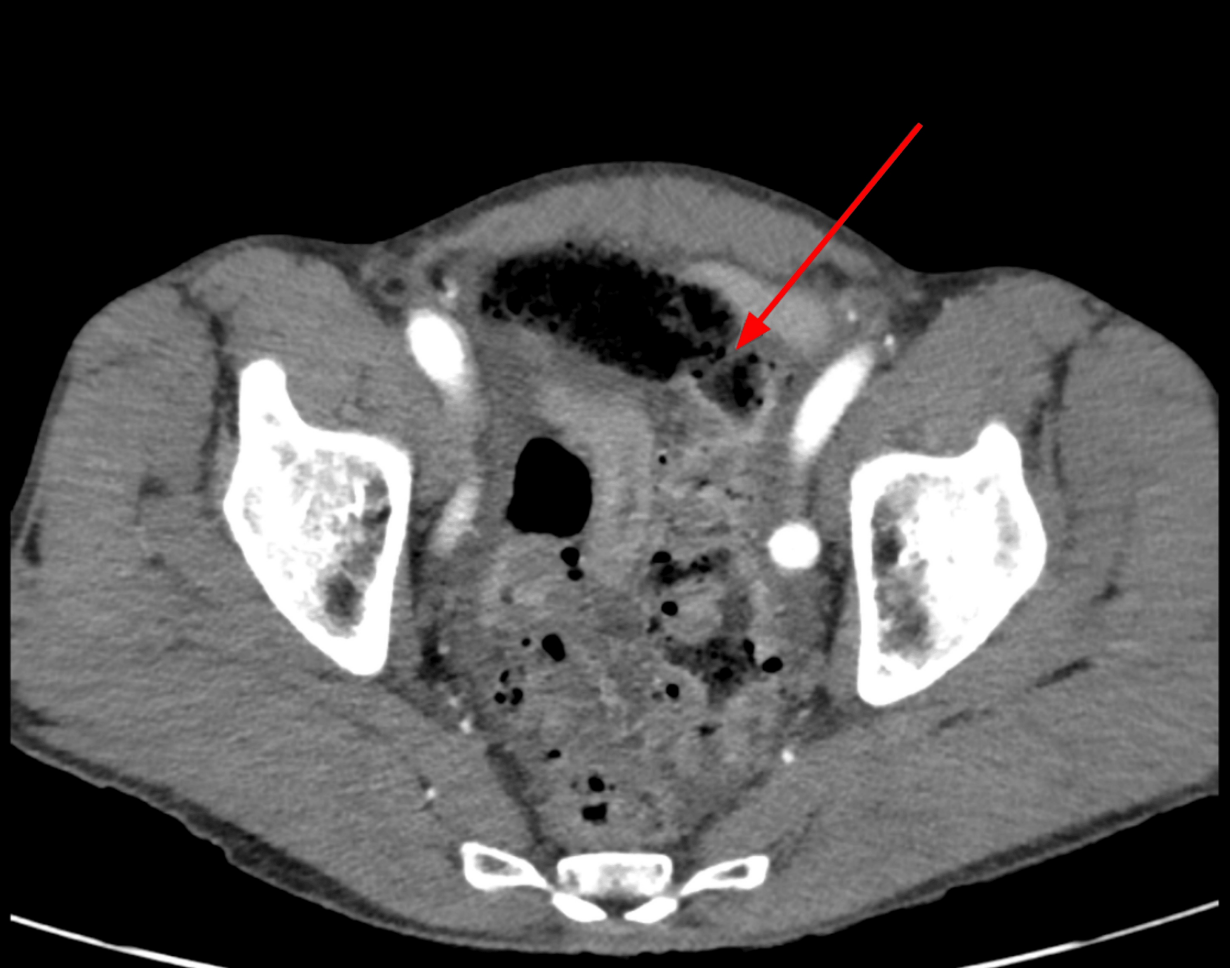
CT in the transverse plane showing a large perforation of the proximal sigmoid colon (red arrow) with feculent material extending into the anterior pelvis

**Figure 2:**
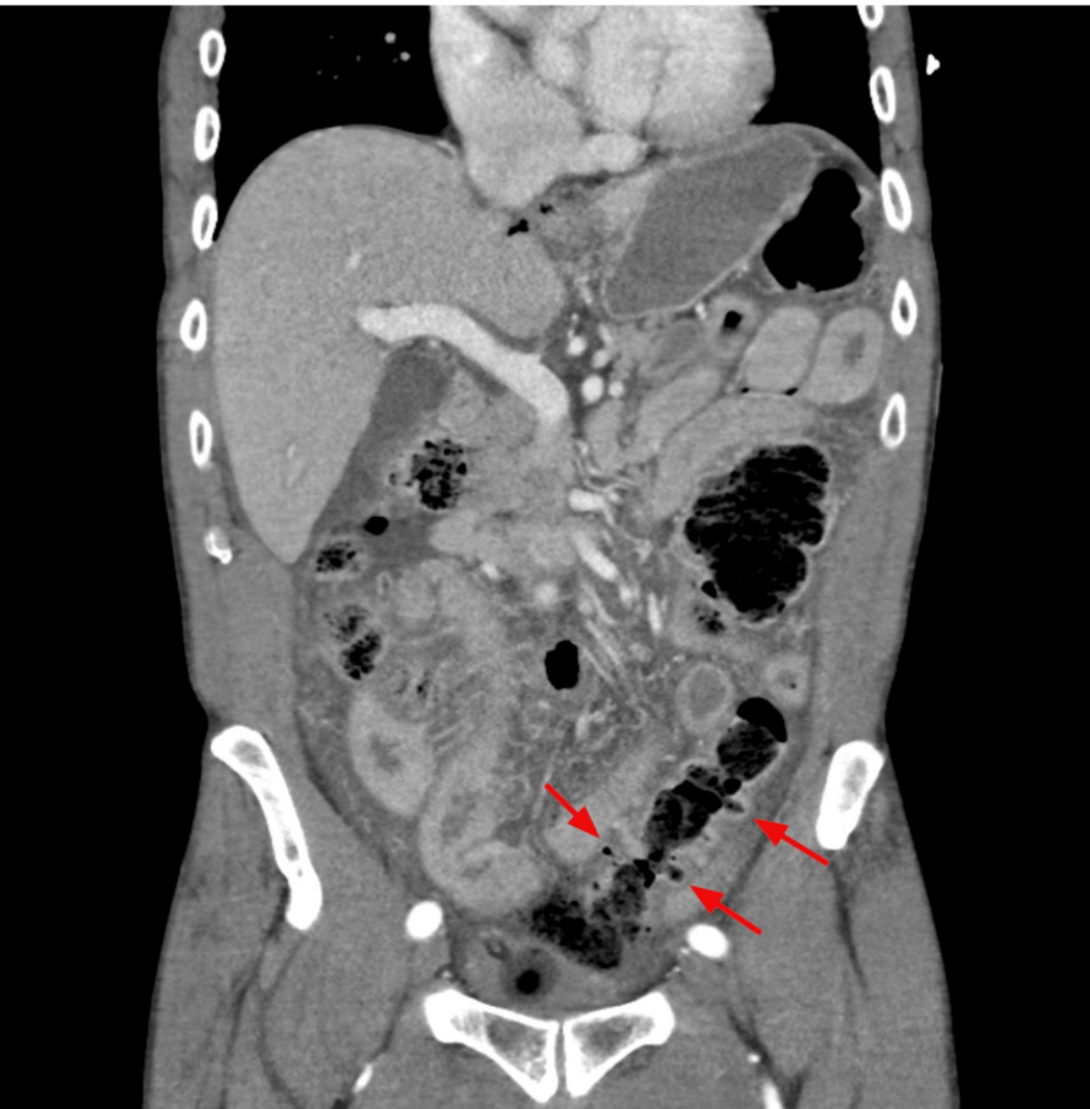
CT in the coronal view showing extensive colonic diverticular disease (red arrows)

A 69-year-old Caucasian male patient with a past medical history of benign prostatic hyperplasia treated with tamsulosin, finasteride, and gabapentin presented to the emergency department via ambulance with a chief complaint of severe abdominal pain. Approximately four hours before arrival, the patient was on his way to use the restroom when he experienced a sharp, crippling abdominal pain that had continued without relief. On initial physical examination, the patient was diaphoretic, bradycardic, and audibly groaning. His abdominal exam revealed hypoactive bowel sounds, a firm and distended abdomen, and diffuse tenderness with guarding. Laboratory investigations revealed a normal white blood cell count of 5.4 x 10^3^/mm^3^ (reference range: 4.5-11 x 10^3^/mm^3^), but a decreased hemoglobin level of 12.5 g/dL (reference range: 13.5-17.5 g/dL). Urine drug screening was positive for opiates and marijuana; however, he did receive morphine approximately 15 minutes prior to testing, and it remains uncertain whether paramedics administered any pain medication. A computed tomography (CT) scan of the abdomen and pelvis with intravenous contrast (Figures 1, 2) revealed a large perforation of the proximal sigmoid colon with feculent material extending into the anterior pelvis with a background of diverticular disease.

The need for emergent surgery was discussed with the patient, who provided informed consent and subsequently underwent an exploratory laparotomy. Upon opening the peritoneum, we encountered foul-smelling, gray-tinged fluid. As the peritoneal defect was widened, both formed and unformed feces were observed throughout the lower abdomen and pelvic region. The abdomen was thoroughly explored, revealing a perforation in the sigmoid colon, along with an extensive stool burden extending from the transverse colon through the entirety of the sigmoid colon. The stool throughout the distal colon was heavily compacted and difficult to remove. These findings confirmed a diagnosis of Hinchey stage IV sigmoid diverticulitis, likely secondary to chronic constipation. The perforated segment of the sigmoid colon was resected and sent to pathology, followed by an extensive washout of the abdomen and pelvis. Due to extensive fecal contamination of the abdomen, the patient was left in discontinuity for a planned second-look laparotomy and subsequent creation of end colostomy, which was performed three days later. Gross examination of the resected specimen revealed a defect measuring 2.1 cm at its greatest dimension, with multiple surrounding intact diverticula. Postoperatively, the patient recovered appropriately, remaining hospitalized for nine days before being discharged for follow-up. At a follow-up appointment, the patient admitted to long-term daily kratom abuse, which he believed contributed to his chronic constipation and subsequent bowel perforation. The duration of his kratom abuse and the exact formulation of kratom which he used are unknown. He had been using kratom primarily for pain management; however, it is unclear whether this was in the context of prior opioid use or withdrawal, as he denied opioid use despite testing positive for them upon initial presentation to the emergency department. Approximately three months after the initial operation, the patient underwent a robotic colostomy reversal. His postoperative course was uneventful, and he was discharged without complaints.

## Discussion

Kratom’s increasing popularity in the United States raises significant concerns regarding its adverse effects, potential for abuse, and lack of regulatory oversight. Although rare, Hinchey stage IV diverticulitis is a life-threatening surgical emergency that, in this patient, may have been preventable. Given his history of long-standing constipation, it is likely that kratom use contributed to the development of his bowel perforation [12]. We do recognize that the patient tested positive for opioids on his initial presentation to the hospital. However, given the patient’s denial of opioid use and his unprompted confession of long-term kratom abuse, we believe kratom to be the primary source of his constipation.

This case underscores the critical need for healthcare providers to routinely inquire about herbal and dietary supplement use. Many patients do not consider herbal products to be medications and may perceive them as inherently safe due to their natural origins. As a result, they may not voluntarily disclose their use unless specifically asked [20]. In this instance, the patient did not admit to using kratom until several weeks after his initial hospitalization. Increased awareness and education regarding kratom and other herbal supplements can enhance patient safety by facilitating early recognition of potential complications.

A key limitation of this case is the inability to establish a definitive causal relationship between kratom use and the patient’s bowel perforation. While the patient’s history of chronic kratom use and associated constipation suggests a plausible link, other contributing factors such as age, diet, comorbidities, and underlying diverticulosis cannot be ruled out. Additionally, the delay in disclosing kratom use limited our ability to assess serum or urine levels at presentation.

The lack of federal regulation surrounding kratom remains a significant public health concern [18]. While kratom may have some legitimate therapeutic applications, its safety profile remains poorly understood, and its unregulated status allows for inconsistencies in potency and purity [19]. Implementing federal regulations on kratom, including standardization of potency, purity testing, and clear labeling of potential risks, would help mitigate harm and improve consumer safety. Until such measures are in place, clinicians should caution patients about the potential dangers of kratom use and remain vigilant for its associated complications.

## Conclusions

Given the increasing prevalence of kratom use, it is essential for clinicians to recognize its effects, clinical presentation, and potential toxicities. A kratom user's pursuit of pain relief may inadvertently lead to severe complications, as demonstrated in this case. Although prompt surgical management is essential for Hinchey stage IV diverticulitis, clinicians should consider kratom use among the possible risk factors during postoperative evaluation and counseling. Further research is needed to clarify the gastrointestinal risks associated with chronic kratom consumption. Until then, patients should be cautioned about its potential complications to help prevent outcomes like those seen in this case.
